# Short- and long-term impact of cancer on employment and financial outcomes of adolescents and young adults (AYAs): a large population-based case-control registry study in the Netherlands

**DOI:** 10.1016/j.esmoop.2022.100521

**Published:** 2022-06-27

**Authors:** S.H.M. Janssen, D.J. van der Meer, M.C.H.J. van Eenbergen, E. Manten-Horst, W.T.A. van der Graaf, O. Husson

**Affiliations:** 1 Department of Psychosocial Research and Epidemiology, Netherlands Cancer Institute, Amsterdam, the Netherlands; 2 Department of Medical Oncology, Netherlands Cancer Institute – Antoni van Leeuwenhoek, Amsterdam, the Netherlands; 3 Department of Research and Development, Netherlands Comprehensive Cancer Organization, Utrecht, the Netherlands; 4 Department of Communication and Cognition, Tilburg University, Tilburg, the Netherlands; 5 Dutch AYA Care Network, Utrecht, the Netherlands; 6 Department of Medical Oncology, Erasmus MC Cancer Institute, Erasmus University Medical Center, Rotterdam, the Netherlands; 7 Department of Surgical Oncology, Erasmus MC Cancer Institute, Erasmus University Medical Center, Rotterdam, the Netherlands

**Keywords:** adolescents and young adults, AYAs, cancer, survivorship, employment and financial outcomes

## Abstract

**Background:**

Adolescent and young adult (AYA) cancer survivors, 18-39 years at initial cancer diagnosis, often self-report negative consequences of cancer (treatment) for their career. Less is known, however, about the objective impact of cancer on employment and financial outcomes. This study examines the employment and financial outcomes of AYA cancer survivors with nationwide population-based registry data and compares the outcomes of AYAs with cancer with an age- and sex-matched control population at year of diagnosis, 1 year later (short-term) and 5 years later (long-term).

**Patients and methods:**

A total of 2527 AYAs, diagnosed in 2013 with any invasive tumor type and who survived for 5 years, were identified from the Netherlands Cancer Registry (clinical and demographic data) and linked to Statistics Netherlands (demographic, employment and financial data). AYAs were matched 1 : 4 with a control population based on age and sex (10 108 controls). Analyses included descriptive statistics, chi-square tests, independent samples *t*-tests, McNemar tests and logistic regression.

**Results:**

AYA cancer survivors were significantly less often employed compared with their controls 1 year (76.1% versus 79.5%, *P* < 0.001) and 5 years (79.3% versus 83.5%, *P* < 0.001) after diagnosis, and received more often disability benefits (9.9% versus 3.1% 1 year after diagnosis, *P* < 0.001; 11.2% versus 3.8% 5 years after diagnosis, *P* < 0.001). Unemployed AYAs were more often diagnosed with higher disease stages (*P* < 0.001), treated with chemotherapy (*P* < 0.001), radiotherapy (*P* < 0.001) or hormone therapy (*P* < 0.05) and less often with local surgery (*P* < 0.05) compared with employed AYAs 1 and 5 years after diagnosis.

**Conclusion:**

Based on objective, nationwide, population-based registry data, AYAs’ employment and financial outcomes are significantly affected compared with age- and sex-matched controls, both short and long-term after cancer diagnosis. Providing support regarding employment and financial outcomes from diagnosis onwards may help AYAs finding their way (back) into society.

## Highlights

• Based on objective data, AYAs’ employment and financial outcomes are significantly affected compared with matched controls.
• AYAs were significantly more often unemployed compared with their controls 1 and 5 years after diagnosis.
• AYAs received significantly more often disability benefits compared with their controls 1 and 5 years after diagnosis.

## Introduction

Over the past three decades, overall cancer incidence among adolescents and young adults (AYAs, aged 18-39 years at initial diagnosis) has increased and with an overall 5-year relative survival of >85%, most AYA cancer survivors have a long life ahead of them.^1^^,^^2^ Adolescence and young adulthood are characterized by physical, cognitive, emotional and social transitions in which AYAs aim to achieve developmental milestones, like finding a job, becoming (financially) independent, forming relationships and starting a family.^3^ A cancer diagnosis, treatment and subsequent physical and psychosocial issues may interrupt and delay or even impede the achievement of these personal goals both in the short and long-term.^4^, ^5^, ^6^, ^7^, ^8^, ^9^

Employment is considered a key aspect of healthy AYA cancer survivorship. It enables AYAs to regain a sense of normalcy (i.e. structure), to recover their sense of identity and role in society with a range of positive consequences for their overall health and health-related quality of life (hrQoL) and provides AYAs with an income to become fully independent.^10^, ^11^, ^12^, ^13^, ^14^ As AYAs are all of working age with possibly many years of employment to follow and overall high survival rates, being employed is of importance for both the individual as well as for society.^15^ Unfortunately, many AYAs with cancer report undesired consequences of cancer (treatment) for their career, including unemployment*,* adverse employment changes (resulting in reduced income), career reorientation and a need to apply for disability benefits.^6^^,^^16^, ^17^, ^18^, ^19^, ^20^, ^21^, ^22^ Although the majority of AYAs eventually return to work, studies suggest lower ratings of employed AYAs compared with the general population.^16^^,^^23^^,^^24^ As AYAs are at the start of their working career with possibly few or even no years of experience and often on temporary contracts, (re)integration into employment may be very challenging for them and may hinder AYAs from becoming financially independent and autonomous.^23^^,^^25^, ^26^, ^27^, ^28^ This can affect the patient’s family as well and may lead to distress and lower hrQoL.^6^^,^^20^^,^^23^^,^^26^, ^27^, ^28^, ^29^, ^30^, ^31^ It is unknown whether the social security system in the Netherlands, where one can apply for benefits in case of unemployment and (partial) disability, covers the possible negative consequences of a cancer diagnosis at adolescence or young adulthood.

Up until now, most research on the impact of cancer on the employment of AYA cancer survivors used self-reported instead of objective data measures. Although self-reported data will provide a good overview of the experiences of AYAs, these data are prone to recall bias and socially desirable answers.^32^ Understanding the objective employment and financial outcomes of AYA cancer survivors over time provides insight into who is at risk of poor outcomes and provides input for the development of relevant services and resources to serve those at risk. The aims of this nationwide population-based registry study are to (i) describe the baseline, short-term (defined as 1 year after diagnosis) and long-term (defined as 5 years after diagnosis) employment and financial outcomes of a Dutch cohort of AYA cancer survivors, (ii) compare these with the employment and financial outcomes of age- and sex-matched controls and (iii) examine factors associated with both short- and long-term employment among the AYAs. In this paper, employment comprises all forms of paid work (e.g. employee, self-employed) and does not include unpaid volunteering.

## Methods

### Data collection

The present study used objective, nationwide, population-based registry data from the Netherlands Cancer Registry (NCR) and Statistics Netherlands (CBS). Since 1989, the NCR obtains disease- and treatment-specific data on all cancer patients in the Netherlands.^33^ CBS systematically collects data on social and economic themes from all people in the Netherlands.^34^

AYA cancer survivors diagnosed in 2013 with their first invasive tumor of any type were selected from the NCR: all AYAs were between the age of 18 and 39 at diagnosis and survived for at least 5 years with no second cancer diagnosis. The NCR sent the AYAs’ demographic and clinical data (see Measures) to CBS, who linked the AYAs to their unique CBS identification number (ID), based on their 6-digit postal code, date of birth and sex. Then, the records were enriched with data from selected CBS registries on demographic characteristics [sex, date of birth (month and year), migration background, educational level, household and marital status] and employment and financial outcomes, based on their ID.^35^ Linkage was carried out for the years 2013 (baseline), 2014 (short-term: 1 year after baseline) and 2018 (long-term: 5 years after baseline). In case of multiple hits per year (multiple jobs at one moment in time), all hits were included in the database. Every case was matched 1 : 4, based on age (year and month of birth) and sex, to compose a control group from all people in the Netherlands based on CBS data.^36^ The control population consisted of people who were alive at baseline and not part of the AYA cancer survivor population. Controls had to be present in the CBS dataset regarding personal income to include those for whom 5 year follow-up was available and were similarly enriched with data from the selected CBS registries as the AYAs. Conditions that needed to be satisfied for all records included presence in the municipal personal records database and in the demographic datasets for all years to avoid inclusion of those who emigrated since 2013. The NCR and CBS both approved linkage, access and utilization of their data and results are based on calculations using non-public microdata from CBS.

### Measures

Demographic characteristics: age at baseline, sex, educational level, marital status, household, migration background.

Employment and financial outcomes: employment (e.g. self-employed, employee, type of contract), benefits, financial (e.g. personal/household income, economic independence).

Clinical characteristics: date of diagnosis and death (if patient died >5 years after diagnosis and before data collection); age at diagnosis; vital status; follow-up in days; invasive tumor type; type of treatment; TNM (tumour–node–metastasis) classification, Figo (gynecological malignancies) or Ann Arbor (hematological malignancies); and type of hospital of treatment.

### Statistical analysis

Statistical analyses were carried out using IBM SPSS Statistics version 25 (SPSS Inc., Chicago, IL). Two-sided *P* values of <0.05 were considered statistically significant. All variables were described as means and standard deviations (continuous data) or frequencies and percentages (categorical data). Independent samples *t*-tests (continuous data) and chi-square tests (categorical data) were carried out to compare (i) those linked with their CBS ID versus those not linked (to test the representativeness of the study sample); (ii) AYA cancer survivors versus the control population; and (iii) employed versus unemployed AYA cancer survivors (to describe differences). McNemar tests were carried out to determine whether there were differences in employment among the AYA cancer survivors over time. Logistic regression analyses were carried out to examine factors associated with employment in the short and long-term among the AYAs. Independent variables with *P* < 0.1 in univariable regression analyses were included in the multivariable logistic regression analyses, except for factors with high multi-collinearity. Tolerance, variance inflation factors and variance proportions were used to test for multi-collinearity. Missing data were not imputed and assumed missing at random.

## Results

**Figure 1:**
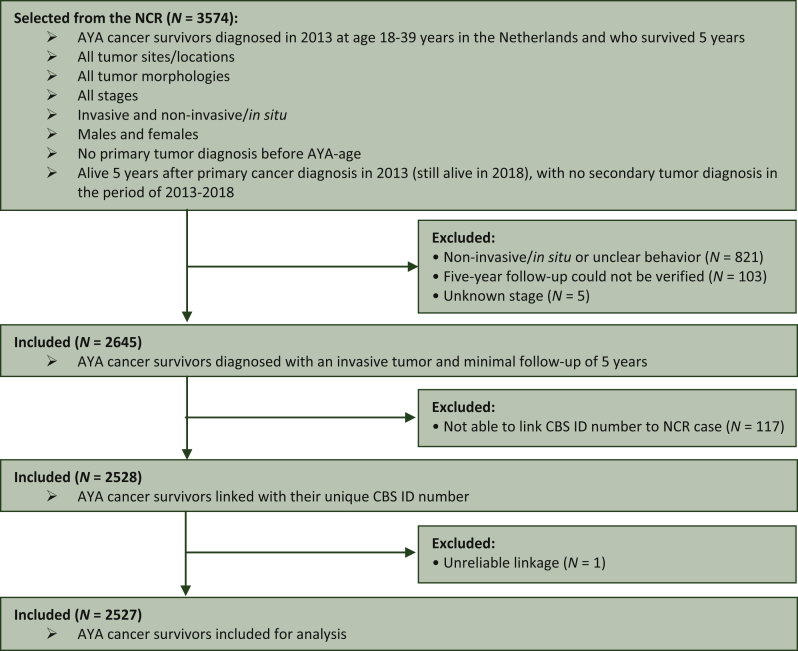
**Flow chart of case selection procedure**. AYA, adolescent and young adult; CBS, Statistics Netherlands; ID, identification; NCR, the Netherlands Cancer Registry.

The NCR provided a cohort of 3574 AYA cancer survivors (Figure 1). Of these, 929 AYAs were excluded, as they did not satisfy the inclusion criteria and 117 AYAs were excluded, because CBS linkage was not possible. Eventually, 2528 AYAs were linked with their CBS ID (95.6% linkage rate). Supplementary Table S1, available at https://doi.org/10.1016/j.esmoop.2022.100521 provides an overview of the characteristics of the AYAs who were linked with their CBS ID and those not. Non-linked AYAs were younger (*P* < 0.001) and received treatment less often (*P* < 0.05, data not shown) compared with those linked with their CBS ID. One AYA was excluded, because of unreliable linkage. Finally, 2527 AYAs were matched (1 : 4) resulting in a control population of 10 108 records.

### AYA cancer survivors versus control population

#### Characteristics of the AYA cancer survivors and control population

Table 1 shows the demographic characteristics of the AYA cancer survivors and their matched controls, and the clinical characteristics of the AYAs. Almost 60% was female, on average 32 years old at baseline and no differences were found based on marital status and educational level. Most common cancer types were breast (20.1%), skin (20.2%) and male genital organ (17.6%).

**Table 1:** Demographic characteristics of the AYA cancer survivors and controls, and clinical characteristics of the AYA cancer survivors

	Baseline		*P* value	One year after baseline (short-term)		*P* value	Five years after baseline (long-term)		*P* value
	AYAs (*N* = 2527)	Controls (*N* = 10 108)		AYAs (*N* = 2527)	Controls (*N* = 10 108)		AYAs (*N* = 2527)	Controls (*N* = 10 108)	
	*N* (%)	*N* (%)		*N* (%)	*N* (%)		*N* (%)	*N* (%)	
Sex									
Male	1068 (42.3)	4272 (42.3)	1.000	—	—	—	—	—	–
Female	1459 (57.7)	5836 (57.7)		—	—		—	—	
Age (at diagnosis) [mean (SD)]	32.2 (5.7)	32.2 (5.7)	1.000		—	—		—	—
Age (at diagnosis)									
18-25	400 (15.8)	1600 (15.8)	1.000	—	—	—	—	—	—
26-39	2127 (84.2)	8508 (84.2)		—	—		—	—	
Country of birth									
The Netherlands	2252 (89.1)	8524 (84.3)	<0.001	—	—	—	—	—	—
Other	275 (10.9)	1584 (15.7)		—	—		—	—	
Migration background^a^									
Suriname	51 (2.0)	276 (2.7)	<0.001	—	—	—	—	—	—
Morocco	56 (2.2)	302 (3.0)		—	—		—	—	
Indonesia	29 (1.1)	114 (1.1)		—	—		—	—	
The Netherlands	2018 (79.9)	7508 (74.3)		—	—		—	—	
Turkey	77 (3.0)	349 (3.5)		—	—		—	—	
Poland	24 (0.9)	129 (1.3)		—	—		—	—	
Other	272 (10.8)	1430 (14.1)		—	—		—	—	
Generation									
Native	2018 (79.9)	7508 (74.3)	<0.001	—	—	—	—	—	—
First-generation immigrant	252 (10.0)	1476 (14.6)		—	—		—	—	
Second-generation immigrant	257 (10.2)	1124 (11.1)		—	—		—	—	
Marital status									
Partner	907 (35.9)	3702 (36.6)	0.494	955 (37.8)	3874 (38.3)	0.621	1124 (44.5)	4419 (43.7)	0.490
No partner	1620 (64.1)	6406 (63.4)		1572 (62.2)	6234 (61.7)		1403 (55.5)	5689 (56.3)	
Educational level (achieved)									
Low^h^	323 (12.8)	1400 (13.9)	0.313	315 (12.5)	1334 (13.2)	0.551	290 (11.5)	1229 (12.2)	0.612
Middle^i^	851 (33.7)	3405 (33.7)		852 (33.7)	3387 (33.5)		800 (31.7)	3160 (31.3)	
High^j^	842 (33.3)	3266 (32.3)		861 (34.1)	3367 (33.3)		967 (38.3)	3823 (37.8)	
Missing	511 (20.2)	2037 (20.2)		499 (19.7)	2020 (20.0)		470 (18.6)	1896 (18.8)	
Educational level (followed)									
Low^h^	169 (6.7)	737 (7.3)	0.332	171 (6.8)	749 (7.4)	0.377	173 (6.8)	736 (7.3)	0.488
Middle^i^	713 (28.2)	2929 (29.0)		710 (28.1)	2878 (28.5)		699 (27.7)	2858 (28.3)	
High^j^	1134 (44.9)	4405 (43.6)		1147 (45.4)	4461 (44.1)		1185 (46.9)	4618 (45.7)	
Missing	511 (20.2)	2037 (20.2)		499 (19.7)	2020 (20.0)		470 (18.6)	1896 (18.8)	
Type of household									
Single person	466 (18.4)	1781 (17.6)	0.217	444 (17.6)	1732 (17.1)	0.067	408 (16.1)	1608 (15.9)	0.004
Couple without children	505 (20.0)	1952 (19.3)		497 (19.7)	1855 (18.4)		461 (18.2)	1589 (15.7)	
Couple with children	1332 (52.7)	5402 (53.4)		1371 (54.3)	5520 (54.6)		1423 (56.3)	5831 (57.7)	
Other^k^	213 (8.4)	972 (9.6)		211 (8.3)	1000 (9.9)		231 (9.1)	1079 (10.7)	
Missing	11 (0.4)	1 (0.0)		4 (0.2)	1 (0.0)		4 (0.2)	1 (0.0)	
Place in household									
Child living at home	371 (14.7)	1462 (14.5)	0.381	336 (13.3)	1255 (12.4)	0.046	182 (7.2)	673 (6.7)	0.005
Single	466 (18.4)	1781 (17.6)		444 (17.6)	1732 (17.1)		408 (16.1)	1608 (15.9)	
Partner without children	502 (19.9)	1931 (19.1)		495 (19.6)	1838 (18.2)		460 (18.2)	1570 (15.5)	
Partner with children	1032 (40.8)	4256 (42.1)		1100 (43.5)	4533 (44.8)		1278 (50.6)	5329 (52.7)	
Parent in one-parent household	89 (3.5)	431 (4.3)		101 (4.0)	514 (5.1)		149 (5.9)	704 (7.0)	
Other^l^	56 (2.2)	246 (2.4)		47 (1.9)	235 (2.3)		46 (1.8)	223 (2.2)	
Missing	11 (0.4)	1 (0.0)		4 (0.2)	1 (0.0)		4 (0.2)	1 (0.0)	
Reference person of household^m^									
No	1404 (55.6)	5658 (56.0)	0.872	1391 (55.0)	5472 (54.1)	0.371	1291 (51.1)	5082 (50.3)	0.425
Yes	1112 (44.0)	4449 (44.0)		1132 (44.8)	4635 (45.9)		1232 (48.8)	5025 (49.7)	
Missing	11 (0.4)	1 (0.0)		4 (0.2)	1 (0.0)		4 (0.2)	1 (0.0)	
Number of persons in household who are living-at-home child(ren)									
0 children	1016 (40.2)	3923 (38.8)	0.150	982 (38.9)	3771 (37.3)	0.135	907 (35.9)	3358 (33.2)	0.010
1-10 child(ren)	1500 (59.4)	6184 (61.2)		1541 (61.0)	6336 (62.7)		1616 (63.9)	6749 (66.8)	
Missing	11 (0.4)	1 (0.0)		4 (0.2)	1 (0.0)		4 (0.2)	1 (0.0)	
Type of cancer									
Bone, articular cartilage and soft tissues	72 (2.8)	—	—	—	—	—	—	—	—
Breast	508 (20.1)	—		—	—		—	—	
Central nervous system	80 (3.2)	—		—	—		—	—	
Digestive tract	120 (4.7)	—		—	—		—	—	
Endocrine glands	124 (4.9)	—		—	—		—	—	
Female genital organs	201 (8.0)	—		—	—		—	—	
Hematological malignancies	339 (13.4)	—		—	—		—	—	
Head and neck	45 (1.8)	—		—	—		—	—	
Male genital organs	444 (17.6)	—		—	—		—	—	
Respiratory tract	23 (0.9)	—		—	—		—	—	
Skin	510 (20.2)	—		—	—		—	—	
Urinary tract	51 (2.0)	—		—	—		—	—	
Other/unspecified sites^b^	10 (0.4)	—		—	—		—	—	
Type of hospital of treatment									
University hospital	645 (25.5)	—	—	—	—	—	—	—	—
General hospital	751 (29.7)	—		—	—		—	—	
Collaborating top clinical hospital	1089 (43.1)	—		—	—		—	—	
Other^c^	42 (1.7)	—		—	—		—	—	
Organ surgery									
No	1148 (45.4)	—	—	—	—	—	—	—	—
Yes	1379 (54.6)	—		—	—		—	—	
Local surgery									
No	1812 (71.7)	—	—	—	—	—	—	—	—
Yes	715 (28.3)	—		—	—		—	—	
Chemotherapy									
No	1471 (58.2)	—	—	—	—	—	—	—	—
Yes	1056 (41.8)	—		—	—		—	—	
Radiotherapy									
No	1781 (70.5)	—	—	—	—	—	—	—	—
Yes	746 (29.5)	—		—	—		—	—	
Hormone therapy									
No	2211 (87.5)	—	—	—	—	—	—	—	—
Yes	316 (12.5)	—		—	—		—	—	
Treatment received^d^									
No	68 (2.7)	—	—	—	—	—	—	—	—
Yes	2459 (97.3)	—		—	—		—	—	
Stage^e^									
I	1172 (59.0)	—	—	—	—	—	—	—	—
II	434 (21.8)	—		—	—		—	—	
III	206 (10.4)	—		—	—		—	—	
IV	47 (2.4)	—		—	—		—	—	
Missing	128 (6.4)	—		—	—		—	—	
Figo stage^f^									
I	162 (80.6)	—	—	—	—	—	—	—	—
II	23 (11.4)	—		—	—		—	—	
III	13 (6.5)	—		—	—		—	—	
IV	3 (1.5)	—		—	—		—	—	
Ann Arbor stage^g^									
I	34 (10.0)	—	—	—	—	—	—	—	—
II	95 (28.0)	—		—	—		—	—	
III	31 (9.1)	—		—	—		—	—	
IV	63 (18.6)	—		—	—		—	—	
Missing	116 (34.2)	—		—	—		—	—	

Independent samples *t*-tests and chi square tests were carried out to compare AYA cancer survivors with the control population.

AYA, adolescent and young adult; SD, standard deviation.

a Countries ≥1% are displayed.

b Unspecified sites, primary sites unknown or unknown tumor type and eye.

c E.g. general practitioner, freely established specialist or foreign hospital.

d Including patients treated with immunotherapy of whom <10 patients received this therapy.

e Figo and Ann Arbor stage were not included in the stage variable.

f Gynecological malignancies.

g Hematological malignancies.

h Primary school, junior high school or equivalent.

i Senior high school or equivalent.

j College/university.

k Institutional households, one-parent household or any other household than already included.

l Member of institutional household, reference person in other household or any other member than already included.

m Member of the household to whom the positions of the other members of the household are determined.

#### Employment and financial outcomes of the AYA cancer survivors and control population

Table 2 provides an overview of the employment and financial outcomes of the AYA cancer survivors and their matched controls at baseline, short-term and long-term. Both 1 and 5 years after diagnosis, AYA cancer survivors were significantly less often employed compared with controls (76.1% versus 79.5%, and 79.3% versus 83.5%, respectively). The number of employed AYAs fluctuated significantly over time from 82.3% at baseline, decreasing to 76.1% 1 year after diagnosis and ending up with 79.3% 5 years after diagnosis (*P* < 0.001). The number of employed controls was more stable in the short-term (79.8% at baseline, 79.5% short-term) and eventually increased in the long-term (83.5%).

**Table 2:** Short- and long-term employment and financial outcomes of the AYA cancer survivors and their matched controls

	Baseline		*P* value	One year after baseline (short-term)		*P* value	Five years after baseline (long-term)		*P* value
	AYAs (*N* = 2527)	Controls (*N* = 10 108)		AYAs (*N* = 2527)	Controls (*N* = 10 108)		AYAs (*N* = 2527)	Controls (*N* = 10 108)	
	*N* (%)	*N* (%)		*N* (%)	*N* (%)		*N* (%)	*N* (%)	
Employed^a^									
No	440 (17.4)	2037 (20.2)	0.002	601 (23.8)	2072 (20.5)	<0.001	522 (20.7)	1669 (16.5)	<0.001
Yes	2079 (82.3)	8071 (79.8)		1924 (76.1)	8036 (79.5)		2005 (79.3)	8438 (83.5)	
Missing	8 (0.3)	—		2 (0.1)	—		—	1 (0.0)	
Employee									
No	638 (25.2)	2875 (28.4)	0.002	810 (32.1)	3002 (29.7)	0.020	777 (30.7)	2846 (28.2)	0.010
Yes	1881 (74.4)	7233 (71.6)		1715 (67.9)	7106 (70.3)		1750 (69.3)	7261 (71.8)	
Missing	8 (0.3)	—		2 (0.1)	—		—	1 (0.0)	
Self-employed									
No	2321 (91.8)	9248 (91.5)	0.294	2316 (91.7)	9148 (90.5)	0.058	2272 (89.9)	8907 (88.1)	0.012
Yes	198 (7.8)	860 (8.5)		209 (8.3)	960 (9.5)		255 (10.1)	1200 (11.9)	
Missing	8 (0.3)	—		2 (0.1)	—		—	1 (0.0)	
Unemployment benefit									
No	2426 (96.0)	9726 (96.2)	0.837	2470 (97.7)	9643 (95.4)	<0.001	2472 (97.8)	9844 (97.4)	0.222
Yes	93 (3.7)	382 (3.8)		55 (2.2)	465 (4.6)		55 (2.2)	263 (2.6)	
Missing	8 (0.3)	—		2 (0.1)	—		—	1 (0.0)	
Social assistance benefit									
No	2447 (96.8)	9680 (95.8)	0.002	2410 (95.4)	9656 (95.5)	0.857	2424 (95.9)	9660 (95.6)	0.445
Yes	72 (2.8)	428 (4.2)		115 (4.6)	452 (4.5)		103 (4.1)	447 (4.4)	
Missing	8 (0.3)	—		2 (0.1)	—		—	1 (0.0)	
Social security benefit									
No	2438 (96.5)	9781 (96.8)	0.961	2427 (96.0)	9771 (96.7)	0.177	2421 (95.8)	9738 (96.3)	0.199
Yes	81 (3.2)	327 (3.2)		98 (3.9)	337 (3.3)		106 (4.2)	369 (3.7)	
Missing	8 (0.3)	—		2 (0.1)	—		—	1 (0.0)	
Sickness/disability benefit									
No	2448 (96.9)	9811 (97.1)	0.749	2276 (90.1)	9794 (96.9)	<0.001	2244 (88.8)	9720 (96.2)	<0.001
Yes	71 (2.8)	297 (2.9)		249 (9.9)	314 (3.1)		283 (11.2)	387 (3.8)	
Missing	8 (0.3)	—		2 (0.1)	—		—	1 (0.0)	
Student									
No	2197 (86.9)	8820 (87.3)	0.957	2295 (90.8)	9031 (89.3)	0.023	2401 (95.0)	9668 (95.6)	0.162
Yes	322 (12.7)	1288 (12.7)		230 (9.1)	1077 (10.7)		126 (5.0)	439 (4.3)	
Missing	8 (0.3)	—		2 (0.1)	—		—	1 (0.0)	
Personal gross income [mean (SD)]	33 681.23 (26 345.56)	33 313.19 (27 199.77)	0.542	34 644.23 (28 244.51)	34 940.57 (28 512.06)	0.640	41 910.92 (32 365.51)	42 712.21 (33 693.16)	0.282
Economic independence of the person									
Not economically independent	831 (32.9)	3381 (33.4)	0.652	888 (35.1)	3246 (32.1)	0.004	704 (27.9)	2401 (23.8)	<0.001
Economically independent	1659 (65.7)	6607 (65.4)		1610 (63.7)	6726 (66.5)		1801 (71.3)	7577 (75.0)	
Missing	37 (1.5)	120 (1.2)		29 (1.1)	136 (1.3)		22 (0.9)	130 (1.3)	
Indicator person with income									
Without personal income	80 (3.2)	404 (4.0)	0.055	94 (3.7)	422 (4.2)	0.299	86 (3.4)	354 (3.5)	0.792
With personal income	2426 (96.0)	9654 (95.5)		2422 (95.8)	9638 (95.4)		2437 (96.4)	9713 (96.1)	
Missing	21 (0.8)	50 (0.5)		11 (0.4)	48 (0.5)		4 (0.2)	41 (0.4)	
Gross household income [mean (SD)]	74 427.01 (47 271.13)	74 009.69 (50 850.23)	0.709	79 575.93 (63 985.44)	77 314.30 (62 225.57)	0.105	91 115.50 (82 067.50)	89 256.17 (61 527.81)	0.207
Benefit dependence of household									
No benefit dependence of household	1701 (67.3)	6982 (69.1)	0.123	1640 (64.9)	6892 (68.2)	0.001	1725 (68.3)	7525 (74.4)	<0.001
Benefit dependence of household	789 (31.2)	3006 (29.7)		858 (34.0)	3080 (30.5)		780 (30.9)	2453 (24.3)	
Missing	37 (1.5)	120 (1.2)		29 (1.1)	136 (1.3)		22 (0.9)	130 (1.3)	

*Employees only*	Baseline		*P* value	One year after baseline (short-term)		*P* value	Five years after baseline (long-term)		*P* value
	AYAs (*N* = 1881)	Controls (*N* = 7233)		AYAs (*N* = 1715)	Controls (*N* = 7106)		AYAs (*N* = 1750)	Controls (*N* = 7261)	
	*N* (%)	*N* (%)		*N* (%)	*N* (%)		*N* (%)	*N* (%)	
Number of employment hours [mean (SD)]	114.83 (43.31)	114.23 (43.61)	0.592	117.80 (43.60)	115.00 (43.87)	0.018	116.98 (39.32)	118.01 (38.11)	0.315
Type of contract									
Fixed term contract	573 (30.5)	2198 (30.4)	0.968	432 (25.2)	2053 (28.9)	0.003	488 (27.9)	1926 (26.5)	0.198
Permanent contract	1259 (66.9)	4791 (66.2)		1239 (72.2)	4800 (67.5)		1217 (69.5)	5086 (70.0)	
Both contract types	36 (1.9)	143 (2.0)		28 (1.6)	137 (1.9)		26 (1.5)	148 (2.0)	
Missing	13 (0.7)	101 (1.4)		16 (0.9)	116 (1.6)		19 (1.1)	101 (1.4)	
Employment									
Full time	854 (45.4)	3261 (45.1)	0.857	804 (46.9)	3150 (44.3)	0.240	771 (44.1)	3164 (43.6)	0.421
Part time	986 (52.4)	3776 (52.2)		879 (51.3)	3767 (53.0)		945 (54.0)	3907 (53.8)	
Both types of employment	28 (1.5)	95 (1.3)		16 (0.9)	73 (1.0)		15 (0.9)	89 (1.2)	
Missing	13 (0.7)	101 (1.4)		16 (0.9)	116 (1.6)		19 (1.1)	101 (1.4)	

Independent samples *t*-tests and chi-square tests were carried out to compare AYA cancer survivors with the control population.

Frequencies ≤10 could not be displayed due to disclosure guidelines of CBS.

AYA, adolescent and young adult; SD, standard deviation.

a Employment includes employees, self-employed and director-major stakeholders.

Controls were significantly more often employees, both short and long-term, compared with the AYA cancer survivors. Among the employees, no significant differences were seen regarding part-time or full-time employment. AYA employees had significantly more often a permanent contract 1 year after baseline compared with controls. This effect, however, was not observed 5 years after diagnosis (Table 2).

Controls received significantly more often social assistance benefit (i.e. benefit for which one can apply in case of little or no income to pay for necessary costs e.g. food, health insurance, rent) at baseline and unemployment benefits 1 year after baseline compared with the AYA cancer survivors (Table 2). AYA cancer survivors received significantly more often disability benefits compared with controls both short (9.9% versus 3.1%, *P* < 0.001) and long-term (11.2% versus 3.8%, *P* < 0.001). Personal and household income did not significantly differ between AYAs and controls for all time points. Economic independence, defined as the situation in which the personal net income from employment is higher than the net social assistance benefit for a single person, did not differ between AYA cancer survivors and controls at baseline. One and five years later, however, controls were more often economically independent compared with the AYAs. Controls were significantly more often studying 1 year after baseline than AYAs, but no significant difference was seen at baseline or 5 years later.

In contrast to baseline, the survivors’ households were significantly more often dependent on receiving benefits compared with the controls’ households, short- and long-term (Table 2).

### AYA cancer survivors

#### Demographic and clinical characteristics, and employment and financial outcomes of (un)employed AYA cancer survivors

The percentage of unemployed AYAs significantly increases from baseline (17.4%) to 1 year later (23.8%) and still remains increased 5 years after diagnosis (20.7%) (*P* < 0.001). Employed AYAs were more often born in the Netherlands, had more often a partner and completed or followed high education compared with unemployed AYAs for all time points (Table 3). They were also significantly older at baseline (32.5 years versus 30.6 years) and 1 year after baseline (33.7 years versus 31.6 years), although no significant difference was seen 5 years after baseline. Females were significantly more often unemployed in the long-term than males (68.4% versus 31.6%). Unemployed AYAs 1 and 5 years after diagnosis were more often treated with chemotherapy, radiotherapy or hormone therapy, and less often with local surgery. Employed AYAs were significantly more often diagnosed with stage I compared with those unemployed 1 and 5 years after diagnosis.

**Table 3:** Demographic and clinical characteristics, and employment and financial outcomes of (un)employed AYA cancer survivors

	Baseline		*P* value	One year after baseline (short-term)		*P* value	Five years after baseline (long-term)		*P* value
	Employed AYAs (*N* = 2079)	Unemployed AYAs (*N* = 440)		Employed AYAs (*N* = 1924)	Unemployed AYAs (*N* = 601)		Employed AYAs (*N* = 2005)	Unemployed AYAs (*N* = 522)	
	*N* (%)	*N* (%)		*N* (%)	*N* (%)		*N* (%)	*N* (%)	
Sex									
Male	884 (42.5)	179 (40.7)	0.478	831 (43.2)	236 (39.3)	0.089	903 (45.0)	165 (31.6)	<0.001
Female	1195 (57.5)	261 (59.3)		1093 (56.8)	365 (60.7)		1102 (55.0)	357 (68.4)	
Age [mean (SD)]	32.5 (5.5)	30.6 (6.5)	<0.001	33.7 (5.4)	31.6 (6.5)	<0.001	37.2 (5.6)	36.9 (6.1)	0.234
Country of birth									
The Netherlands	1901 (91.4)	350 (79.5)	<0.001	1765 (91.7)	486 (80.9)	<0.001	1839 (91.7)	413 (79.1)	<0.001
Other	178 (8.6)	90 (20.5)		159 (8.3)	115 (19.1)		166 (8.3)	109 (20.9)	
Migration background^a^									
Suriname	34 (1.6)	16 (3.6)	<0.001	29 (1.5)	22 (3.7)	<0.001	34 (1.7)	17 (3.3)	<0.001
Morocco	35 (1.7)	21 (4.8)		24 (1.2)	32 (5.3)		24 (1.2)	32 (6.1)	
Indonesia	24 (1.2)	5 (1.1)		21 (1.1)	8 (1.3)		21 (1.0)	8 (1.5)	
The Netherlands	1730 (83.2)	287 (65.2)		1620 (84.2)	397 (66.1)		1682 (83.9)	336 (64.4)	
Turkey	44 (2.1)	33 (7.5)		36 (1.9)	41 (6.8)		29 (1.4)	48 (9.2)	
Poland	15 (0.7)	9 (2.0)		16 (0.8)	8 (1.3)		17 (0.8)	7 (1.3)	
Other	197 (9.5)	69 (15.7)		178 (9.3)	93 (15.5)		198 (9.9)	74 (14.2)	
Generation									
Native	1730 (83.2)	287 (65.2)	<0.001	1620 (84.2)	397 (66.1)	<0.001	1682 (83.9)	336 (64.4)	<0.001
First-generation immigrant	157 (7.6)	88 (20.0)		140 (7.3)	111 (18.5)		149 (7.4)	103 (19.7)	
Second-generation immigrant	192 (9.2)	65 (14.8)		164 (8.5)	93 (15.5)		174 (8.7)	83 (15.9)	
Marital status									
Partner	773 (37.2)	132 (30.0)	0.004	775 (40.3)	180 (30.0)	<0.001	914 (45.6)	210 (40.2)	0.028
No partner	1306 (62.8)	308 (70.0)		1149 (59.7)	421 (70.0)		1091 (54.4)	312 (59.8)	
Educational level (achieved)									
Low^b^	201 (9.7)	122 (27.7)	<0.001	160 (8.3)	155 (25.8)	<0.001	154 (7.7)	136 (26.1)	<0.001
Middle^c^	658 (31.6)	192 (43.6)		582 (30.2)	270 (44.9)		605 (30.2)	195 (37.4)	
High^d^	775 (37.3)	66 (15.0)		752 (39.1)	109 (18.1)		863 (43.0)	104 (19.9)	
Missing	445 (21.4)	60 (13.6)		430 (22.3)	67 (11.1)		383 (19.1)	87 (16.7)	
Educational level (followed)									
Low^b^	97 (4.7)	72 (16.4)	<0.001	79 (4.1)	92 (15.3)	<0.001	80 (4.0)	93 (17.8)	<0.001
Middle^c^	545 (26.2)	167 (38.0)		480 (24.9)	230 (38.3)		513 (25.6)	186 (35.6)	
High^d^	992 (47.7)	141 (32.0)		935 (48.6)	212 (35.3)		1029 (51.3)	156 (29.9)	
Missing	445 (21.4)	60 (13.6)		430 (22.3)	67 (11.1)		383 (19.1)	87 (16.7)	
Type of household									
Single person	383 (18.4)	83 (18.9)	<0.001	329 (17.1)	115 (19.1)	<0.001	306 (15.3)	102 (19.5)	<0.001
Couple without children	454 (21.8)	51 (11.6)		421 (21.9)	76 (12.6)		386 (19.3)	75 (14.4)	
Couple with children	1109 (53.3)	223 (50.7)		1056 (54.9)	315 (52.4)		1175 (58.6)	248 (47.5)	
Other^e^	132 (6.3)	81 (18.4)		116 (6.0)	95 (15.8)		136 (6.8)	95 (18.2)	
Missing	1 (0.0)	2 (0.5)		2 (0.1)	—		2 (0.1)	2 (0.4)	
Place in household									
Child living at home	267 (12.8)	104 (23.6)	<0.001	200 (10.4)	136 (22.6)	<0.001	127 (6.3)	55 (10.5)	<0.001
Single	383 (18.4)	83 (18.9)		329 (17.1)	115 (19.1)		306 (15.3)	102 (19.5)	
Partner without children	451 (21.7)	51 (11.6)		420 (21.8)	75 (12.5)		385 (19.2)	75 (14.4)	
Partner with children	888 (42.7)	144 (32.7)		899 (46.7)	201 (33.4)		1072 (53.5)	206 (39.5)	
Parent in one-parent household	58 (2.8)	31 (7.0)		55 (2.9)	46 (7.7)		87 (4.3)	62 (11.9)	
Other^f^	31 (1.5)	25 (5.7)		19 (1.0)	28 (4.7)		26 (1.3)	20 (3.8)	
Missing	1 (0.0)	2 (0.5)		2 (0.1)	—		2 (0.1)	2 (0.4)	
Reference person of household^g^									
No	1124 (54.1)	280 (63.6)	<0.001	1026 (53.3)	365 (60.7)	0.002	996 (49.7)	295 (56.5)	0.004
Yes	954 (45.9)	158 (35.9)		896 (46.6)	236 (39.3)		1007 (50.2)	225 (43.1)	
Missing	1 (0.0)	2 (0.5)		2 (0.1)	—		2 (0.1)	2 (0.4)	
Number of persons in household who are living-at-home child(ren)									
0 Children	861 (41.4)	155 (35.2)	0.019	767 (39.9)	215 (35.8)	0.070	713 (35.6)	194 (37.2)	0.469
1-10 Child(ren)	1217 (58.5)	283 (64.3)		1155 (60.0)	386 (64.2)		1290 (64.3)	326 (62.5)	
Missing	1 (0.0)	2 (0.5)		2 (0.1)	—		2 (0.1)	2 (0.4)	
Type of cancer									
Bone, articular cartilage and soft tissues	61 (2.9)	11 (2.5)	n.a.	53 (2.8)	19 (3.2)	n.a.	49 (2.4)	23 (4.4)	n.a.
Breast	420 (20.2)	87 (19.8)		371 (19.3)	137 (22.8)		373 (18.6)	135 (25.9)	
Central nervous system	57 (2.7)	23 (5.2)		47 (2.4)	33 (5.5)		35 (1.7)	45 (8.6)	
Digestive tract	93 (4.5)	25 (5.7)		85 (4.4)	35 (5.8)		98 (4.9)	22 (4.2)	
Endocrine glands	102 (4.9)	22 (5.0)		97 (5.0)	27 (4.5)		105 (5.2)	19 (3.6)	
Female genital organs	156 (7.5)	44 (10.0)		144 (7.5)	56 (9.3)		150 (7.5)	51 (9.8)	
Hematological malignancies	270 (13.0)	66 (15.0)		232 (12.1)	106 (17.6)		257 (12.8)	82 (15.7)	
Head and neck	36 (1.7)	9 (2.0)		33 (1.7)	12 (2.0)		31 (1.5)	14 (2.7)	
Male genital organs	370 (17.8)	74 (16.8)		356 (18.5)	88 (14.6)		389 (19.4)	55 (10.5)	
Respiratory tract	14 (0.7)	9 (2.0)		12 (0.6)	11 (1.8)		13 (0.6)	10 (1.9)	
Skin	451 (21.7)	59 (13.4)		448 (23.3)	62 (10.3)		456 (22.7)	54 (10.3)	
Urinary tract	41 (2.0)	9 (2.0)		39 (2.0)	12 (2.0)		42 (2.1)	9 (1.7)	
Other/unspecified sites^h^	8 (0.4)	2 (0.5)		7 (0.4)	3 (0.5)		7 (0.3)	3 (0.6)	
Type of hospital of treatment									
University hospital	519 (25.0)	122 (27.7)	0.227	464 (24.1)	181 (30.1)	0.003	486 (24.2)	159 (30.5)	0.004
General/collaborating top clinical hospital or other^i^	1560 (75.0)	318 (72.3)		1460 (75.9)	420 (69.9)		1519 (75.8)	363 (69.5)	
Organ surgery									
No	949 (45.6)	195 (44.3)	0.611	878 (45.6)	269 (44.8)	0.707	912 (45.5)	236 (45.2)	0.910
Yes	1130 (54.4)	245 (55.7)		1046 (54.4)	332 (55.2)		1093 (54.5)	286 (54.8)	
Local surgery									
No	1463 (70.4)	341 (77.5)	0.003	1323 (68.8)	487 (81.0)	<0.001	1409 (70.3)	403 (77.2)	0.002
Yes	616 (29.6)	99 (22.5)		601 (31.2)	114 (19.0)		596 (29.7)	119 (22.8)	
Chemotherapy									
No	1219 (58.6)	248 (56.4)	0.380	1177 (61.2)	293 (48.8)	<0.001	1228 (61.2)	243 (46.6)	<0.001
Yes	860 (41.4)	192 (43.6)		747 (38.8)	308 (51.2)		777 (38.8)	279 (53.4)	
Radiotherapy									
No	1474 (70.9)	302 (68.6)	0.344	1398 (72.7)	381 (63.4)	<0.001	1454 (72.5)	327 (62.6)	<0.001
Yes	605 (29.1)	138 (31.4)		526 (27.3)	220 (36.6)		551 (27.5)	195 (37.4)	
Hormone therapy									
No	1824 (87.7)	380 (86.4)	0.430	1700 (88.4)	509 (84.7)	0.018	1776 (88.6)	435 (83.3)	0.001
Yes	255 (12.3)	60 (13.6)		224 (11.6)	92 (15.3)		229 (11.4)	87 (16.7)	
Treatment received									
No	46 (2.2)	20 (4.5)	0.005	43 (2.2)	25 (4.2)	0.011	47 (2.3)	21 (4.0)	0.035
Yes	2033 (97.8)	420 (95.5)		1881 (97.8)	576 (95.8)		1958 (97.7)	501 (96.0)	
Stage^j^									
I	992 (60.0)	179 (54.2)	0.384	966 (62.4)	206 (46.9)	<0.001	1009 (63.1)	163 (41.9)	<0.001
II	364 (22.0)	70 (21.2)		323 (20.9)	111 (25.3)		331 (20.7)	103 (26.5)	
III	163 (9.9)	41 (12.4)		147 (9.5)	59 (13.4)		155 (9.7)	51 (13.1)	
IV	38 (2.3)	8 (2.4)		31 (2.0)	16 (3.6)		29 (1.8)	18 (4.6)	
Missing	96 (5.8)	32 (9.7)		81 (5.2)	47 (10.7)		74 (4.6)	54 (13.9)	
Figo stage^k^									
I	128 (82.1)	34 (77.3)	—	122 (84.7)	39 (69.6)	—	126 (84.0)	36 (70.6)	—
II	17 (10.9)	≤10		13 (9.0)	10 (17.9)		15 (10.0)	≤10	
III	≤10	≤10		≤10	≤10		≤10	≤10	
IV	≤10	≤10		≤10	≤10		≤10	≤10	
Ann Arbor stage^l^									
I	27 (10.0)	≤10	—	21 (9.1)	13 (12.3)	—	29 (11.3)	≤10	—
II	77 (28.5)	18 (27.3)		70 (30.2)	25 (23.6)		79 (30.7)	16 (19.5)	
III	29 (10.7)	≤10		21 (9.1)	10 (9.4)		27 (10.5)	≤10	
IV	52 (19.3)	10 (15.2)		46 (19.8)	17 (16.0)		47 (18.3)	16 (19.5)	
Missing	85 (31.5)	29 (43.9)		74 (31.9)	41 (38.7)		75 (29.2)	41 (50.0)	
Employee									
No	198 (9.5)	—	n.a.	209 (10.9)	—	n.a.	255 (12.7)	—	n.a.
Yes	1881 (90.5)	—		1715 (89.1)	—		1750 (87.3)	—	
Self-employed									
No	1881 (90.5)	—	n.a.	1715 (89.1)	—	n.a.	1750 (87.3)	—	n.a.
Yes	198 (9.5)	—		209 (10.9)	—		255 (12.7)	—	
Unemployment benefit									
No	2042 (98.2)	384 (87.3)	<0.001	1900 (98.8)	570 (94.8)	<0.001	1976 (98.6)	496 (95.0)	<0.001
Yes	37 (1.8)	56 (12.7)		24 (1.2)	31 (5.2)		29 (1.4)	26 (5.0)	
Social assistance benefit									
No	2069 (99.5)	378 (85.9)	<0.001	1912 (99.4)	498 (82.9)	<0.001	1995 (99.5)	429 (82.2)	<0.001
Yes	10 (0.5)	62 (14.1)		12 (0.6)	103 (17.1)		10 (0.5)	93 (17.8)	
Social security benefit									
No	2055 (98.8)	383 (87.0)	<0.001	1883 (97.9)	544 (90.5)	<0.001	1971 (98.3)	450 (86.2)	<0.001
Yes	24 (1.2)	57 (13.0)		41 (2.1)	57 (9.5)		34 (1.7)	72 (13.8)	
Sickness/disability benefit									
No	2060 (99.1)	388 (88.2)	<0.001	1870 (97.2)	406 (67.6)	<0.001	1921 (95.8)	323 (61.9)	<0.001
Yes	19 (0.9)	52 (11.8)		54 (2.8)	195 (32.4)		84 (4.2)	199 (38.1)	
Student									
No	1861 (89.5)	336 (76.4)	<0.001	1796 (93.3)	499 (83.0)	<0.001	1913 (95.4)	488 (93.5)	0.072
Yes	218 (10.5)	104 (23.6)		128 (6.7)	102 (17.0)		92 (4.6)	34 (6.5)	
Personal gross income [mean (SD)]	38 431.45 (26 153.69)	10 618.25 (10 087.29)	<0.001	41 300.21 (28 276.21)	13 107.51 (13 604.81)	<0.001	48 551.31 (32 500.09)	16 332.64 (13 790.74)	<0.001
Economic independence of the person									
Not economically independent	436 (21.0)	395 (89.8)	<0.001	340 (17.7)	548 (91.2)	<0.001	230 (11.5)	474 (90.8)	<0.001
Economically independent	1639 (78.8)	20 (4.5)		1577 (82.0)	33 (5.5)		1769 (88.2)	32 (6.1)	
Missing	4 (0.2)	25 (5.7)		7 (0.4)	20 (3.3)		6 (0.3)	16 (3.1)	
Indicator person with income									
Without personal income	≤10%	80 (18.2)	<0.001	≤10%	94 (15.6)	<0.001	≤10%	86 (16.5)	<0.001
With personal income	≥90%	348 (79.1)		≥90%	500 (83.2)		≥90%	434 (83.1)	
Missing	1 (0.0)	12 (2.7)		2 (0.1)	7 (1.2)		2 (0.1)	2 (0.4)	
Gross household income [mean (SD)]	79 121.89 (45 120.10)	51 632.69 (50 778.12)	<0.001	85 712.75 (61 352.69)	59 719.09 (68 212.80)	<0.001	99 471.44 (87 287.60)	58 929.07 (45 060.78)	<0.001
Benefit dependence of household									
No benefit dependence of household	1542 (74.2)	159 (36.1)	<0.001	1477 (76.8)	163 (27.1)	<0.001	1601 (79.9)	124 (23.8)	<0.001
Benefit dependence of household	533 (25.6)	256 (58.2)		440 (22.9)	418 (69.6)		398 (19.9)	382 (73.2)	
Missing	4 (0.2)	25 (5.7)		7 (0.4)	20 (3.3)		6 (0.3)	16 (3.1)	

*Employees only*	Baseline		*P* value	One year after baseline (short-term)		*P* value	Five years after baseline (long-term)		*P* value
	Employed AYAs (*N* = 1881)	Unemployed AYAs (*N* = 0)		Employed AYAs (*N* = 1715)	Unemployed AYAs (*N* = 0)		Employed AYAs (*N* = 1750)	Unemployed AYAs (*N* = 0)	
	*N* (%)	*N* (%)		*N* (%)	*N* (%)		*N* (%)	*N* (%)	
Number of employment hours [mean (SD)]	114.83 (43.31)	—	n.a.	117.80 (43.60)	—	n.a.	116.98 (39.32)	—	n.a.
Type of contract									
Fixed-term contract	573 (30.5)	—	n.a.	432 (25.2)	—	n.a.	488 (27.9)	—	n.a.
Permanent contract	1259 (66.9)	—		1239 (72.2)	—		1217 (69.5)	—	
Both contract types	36 (1.9)	—		28 (1.6)	—		26 (1.5)	—	
Missing	13 (0.7)	—		16 (0.9)	—		19 (1.1)	—	
Employment									
Full time	854 (45.4)	—	n.a.	804 (46.9)	—	n.a.	771 (44.1)	—	n.a.
Part time	986 (52.4)	—		879 (51.3)	—		945 (54.0)	—	
Both types of employment	28 (1.5)	—	—	16 (0.9)	—	—	15 (0.9)	—	—
Missing	13 (0.7)	—		16 (0.9)	—		19 (1.1)	—	

Independent samples *t*-tests and chi-square tests were carried out to compare AYA cancer survivors employed and unemployed.

AYA, adolescent and young adult; SD, standard deviation; n.a., not applicable due to low numbers.

a Countries ≥1% are displayed.

b Primary school, junior high school or equivalent.

c Senior high school or equivalent.

d College/university.

e Institutional households, one-parent household or any other household than already included.

f Member of institutional household, reference person in other household or any other member than already included.

g Member of the household to whom the positions of the other members of the household are determined.

h Unspecified sites, primary sites unknown or unknown tumor type and eye.

i For example general practitioner, freely established specialist or foreign hospital.

j Figo and Ann Arbor stage were not included in the stage variable.

k Gynecological malignancies.

l Hematological malignancies.

For all time points, unemployed AYAs received significantly more often unemployment benefits, social assistance benefits, social security benefits and disability benefits compared with those employed (Table 3). Furthermore, unemployed AYAs were significantly less often economically independent compared with those employed: 4.5% versus 78.8% at baseline, 5.5% versus 82.0% 1 year after diagnosis and 6.1% versus 88.2% 5 years after baseline, respectively. Personal income was significantly less among those unemployed for all time points compared with employed AYAs. Also, unemployed AYAs were significantly more often studying compared with employed AYAs at baseline and 1 year later, however, this effect did not withstand 5 years later.

On a household level, the income was on average significantly higher of employed AYAs compared with those unemployed for all time points. At baseline, 58.2% of the households of those unemployed were dependent on receiving benefits, compared with 25.6% of the households of those employed. This significant difference increased 1 year later (69.6% versus 22.9%) and 5 years later (73.2% versus 19.9%).

#### Factors associated with employment

Results of the logistic regression analyses are shown in Table 4. Among the AYAs, employment in the short-term was associated with being of older age and having completed middle- or high-level education. In the long-term, being employed was associated with not having a partner, having completed middle- or high-level education, one’s place in a household and being the reference person of a household. Clinical factors associated with unemployment include a cancer diagnosis of the central nervous system (CNS) (short- and long-term) and being treated with chemotherapy (long-term).

**Table 4:** Logistic regression models evaluating factors associated with short- and long-term employment

	One year after baseline (short-term)				Five years after baseline (long-term)			
	Univariable logistic regression		Multivariable logistic regression		Univariable logistic regression		Multivariable logistic regression	
			Nagelkerkes *R*^2^ = 0.253				Nagelkerkes *R*^2^ = 0.276	
	OR (95% CI)	*P* value	OR (95% CI)	*P* value	OR (95% CI)	*P* value	OR (95% CI)	*P* value
Sex								
Male	Reference		Reference		Reference		Reference	
Female	0.85 (0.705-1.025)	0.089	0.801 (0.538-1.194)	0.276	0.564 (0.46-0.692)	<0.001	0.653 (0.404-1.054)	0.081
Age	1.065 (1.048-1.082)	<0.001	1.029 (1.002-1.057)	0.033	1.011 (0.994-1.028)	0.214	—k	—
Country of birth								
Netherlands	Reference		Reference		Reference		Reference	
Other	0.381 (0.294-0.494)	<0.001	1.289 (0.375-4.431)	0.687	0.342 (0.263-0.445)	<0.001	0.743 (0.228-2.424)	0.623
Generation								
Native	Reference		Reference		Reference		Reference	
First-generation immigrant	0.309 (0.235-0.406)	<0.001	0.401 (0.112-1.441)	0.161	0.289 (0.219-0.381)	<0.001	0.772 (0.226-2.629)	0.678
Second-generation immigrant	0.432 (0.328-0.57)	<0.001	0.524 (0.373-0.737)	<0.001	0.419 (0.314-0.558)	<0.001	0.526 (0.355-0.759)	0.001
Marital status								
Partner	Reference		Reference		Reference		Reference	
No partner	0.634 (0.521-0.772)	<0.001	1.003 (0.710-1.416)	0.987	0.803 (0.661-0.977)	0.028	1.544 (1.077-2.213)	0.018
Educational level (achieved)								
Low^a^	Reference		Reference		Reference		Reference	
Middle^b^	2.088 (1.604-2.719)	<0.001	1.75 (1.148-2.669)	0.009	2.74 (2.068-3.631)	<0.001	2.519 (1.818-3.49)	<0.001
High^c^	6.683 (4.958-9.009)	<0.001	5.467 (3.137-9.529)	<0.001	7.328 (5.388-9.967)	<0.001	6.13 (4.295-8.75)	<0.001
Educational level (followed)								
Low^a^	Reference		Reference		Reference		Reference^l^	
Middle^b^	2.43 (1.731-3.412)	<0.001	1.308 (0.789-2.169)	0.298	3.206 (2.276-4.517)	<0.001	—	—
High^c^	5.136 (3.672-7.184)	<0.001	1.061 (0.573-1.965)	0.851	7.668 (5.441-10.806)	<0.001	—	—
Type of household								
Single person	Reference		Reference		Reference		Reference^l^	
Couple without children	1.936 (1.401-2.676)	<0.001	0.401 (0.023-7.026)	0.531	1.716 (1.229-2.395)	0.002	—	—
Couple with children	1.172 (0.916-1.5)	0.208	0.306 (0.097-0.965)	0.043	1.579 (1.215-2.054)	0.001	—	—
Other^d^	0.427 (0.302-0.602)	<0.001	0.503 (0.195-1.295)	0.154	0.477 (0.338-0.674)	<0.001	—	—
Place in household								
Single	Reference		Reference		Reference		Reference	
Child living at home	0.514 (0.379-0.697)	<0.001	3.349 (0.307-36.555)	0.322	0.77 (0.522-1.134)	0.186	2.564 (1.181-5.566)	0.017
Partner without children	1.957 (1.415-2.708)	<0.001	5.510 (0.319-95.046)	0.240	1.711 (1.226-2.389)	0.002	3.616 (2.115-6.183)	<0.001
Partner with children	1.563 (1.203-2.031)	0.001	5.777 (0.533-62.586)	0.149	1.735 (1.325-2.27)	<0.001	4.697 (2.718-8.119)	<0.001
Parent in one-parent household	0.418 (0.268-0.652)	<0.001	1.050 (0.081-13.538)	0.970	0.468 (0.315-0.695)	<0.001	0.634 (0.374-1.074)	0.09
Other^e^	0.237 (0.128-0.441)	<0.001	—	—	0.433 (0.232-0.809)	0.009	2.03 (0.774-5.32)	0.15
Reference person of household								
No	Reference		Reference		Reference		Reference	
Yes	1.351 (1.121-1.628)	0.002	1.418 (0.88-2.285)	0.152	1.326 (1.091-1.61)	0.004	2.382 (1.384-4.099)	0.002
Number of persons in household who are living-at-home child(ren)								
0 children	Reference		Reference		Reference		Reference^j^	
1-10 child(ren)	0.839 (0.694-1.014)	0.07	1.119 (0.083-15.011)	0.932	1.077 (0.882-1.315)	0.469	—	—
Type of cancer								
Breast	Reference		Reference		Reference		Reference	
Digestive tract	0.897 (0.578-1.392)	0.627	0.628 (0.311-1.265)	0.192	1.612 (0.9750-2.665)	0.063	1.507 (0.675-3.364)	0.317
Respiratory tract	0.403 (0.174-0.934)	0.034	0.414 (0.126-1.357)	0.146	0.471 (0.202-1.098)	0.081	0.377 (0.114-1.244)	0.109
Skin	2.668 (1.918-3.712)	<0.001	1.25 (0.573-2.727)	0.575	3.056 (2.167-4.311)	<0.001	1.823 (0.814-4.082)	0.144
Bone, articular cartilage and soft tissues	1.03 (0.589-1.802)	0.917	0.95 (0.389-2.322)	0.910	0.771 (0.452-1.314)	0.339	0.5 (0.2-1.248)	0.138
Head and neck	1.015 (0.51-2.023)	0.965	1.183 (0.448-3.124)	0.734	0.801 (0.414-1.552)	0.512	0.467 (0.177-1.233)	0.124
Female genital organs	0.95 (0.659-1.369)	0.781	0.787 (0.432-1.433)	0.433	1.065 (0.733-1.547)	0.743	1.075 (0.578-1.999)	0.819
Male genital organs	1.494 (1.102-2.026)	0.010	0.898 (0.474-1.702)	0.742	2.56 (1.814-3.612)	<0.001	0.953 (0.478-1.899)	0.891
Urinary tract	1.2 (0.61-2.36)	0.597	1.144 (0.423-3.089)	0.791	1.689 (0.801-3.563)	0.169	0.754 (0.274-2.078)	0.585
Hematological malignancies	0.808 (0.598-1.093)	0.167	0.694 (0.388-1.242)	0.219	1.134 (0.826-1.558)	0.436	0.97 (0.531-1.771)	0.92
Endocrine glands	1.327 (0.83-2.121)	0.238	1.482 (0.715-3.073)	0.29	2 (1.181-3.387)	0.010	1.202 (0.548-2.634)	0.647
Central nervous system	0.526 (0.323-0.855)	0.010	0.39 (0.17-0.895)	0.026	0.282 (0.174-0.457)	<0.001	0.147 (0.061-0.352)	<0.001
Other^f^	0.862 (0.22-3.379)	0.831	n.s.	n.s.	0.845 (0.215-3.313)	0.809	n.s.	n.s.
Type of hospital of treatment								
University hospital	Reference		Reference		Reference		Reference	
General hospital	1.247 (0.98-1.585)	0.072	0.97 (0.696-1.353)	0.859	1.391 (1.077-1.796)	0.011	0.95 (0.659-1.368)	0.781
Collaborating top clinical hospital	1.369 (1.095-1.712)	0.006	1.198 (0.881-1.629)	0.249	1.33 (1.053-1.679)	0.016	0.95 (0.679-1.33)	0.765
Other^g^	n.s.	n.s.	n.s.	n.s.	2.421 (0.935-6.266)	0.068	0.388 (0.128-1.174)	0.094
Organ surgery								
No	Reference		Reference^j^		Reference		Reference^j^	
Yes	0.965 (0.803-1.16)	0.707	—	—	0.989 (0.815-1.2)	0.91	—	—
Local surgery								
No	Reference		Reference		Reference		Reference	
Yes	1.941 (1.549-2.432)	<0.001	1.299 (0.751-2.248)	0.349	1.432 (1.143-1.795)	0.002	0.79 (0.447-1.398)	0.418
Chemotherapy								
No	Reference		Reference		Reference		Reference	
Yes	0.604 (0.502-0.726)	<0.001	0.836 (0.574-1.218)	0.351	0.551 (0.454-0.669)	<0.001	0.539 (0.356-0.817)	0.004
Radiotherapy								
No	Reference		Reference		Reference		Reference	
Yes	0.652 (0.537-0.791)	<0.001	0.739 (0.543-1.005)	0.054	0.635 (0.519-0.778)	<0.001	1.028 (0.74-1.427)	0.870
Hormone therapy								
No	Reference		Reference		Reference		Reference	
Yes	0.729 (0.561-0.947)	0.018	0.938 (0.581-1.516)	0.794	0.645 (0.493-0.843)	0.001	1.282 (0.783-2.092)	0.319
Immunotherapy								
No	Reference		Reference		Reference		Reference	
Yes	n.s.	n.s.	—	—	n.s.	n.s.	—	—
Treatment received								
No	Reference		Reference		Reference		Reference	
Yes	1.899 (1.15-3.136)	0.012	1.264 (0.554-2.885)	0.578	1.746 (1.034-2.948)	0.037	1.402 (0.543-3.621)	0.485
Stage^h^								
I	Reference		Reference		Reference		Reference	
II	0.621 (0.477-0.807)	<0.001	0.975 (0.672-1.416)	0.895	0.519 (0.394-0.684)	<0.001	0.982 (0.658-1.464)	0.927
III	0.531 (0.379-0.745)	<0.001	0.867 (0.533-1.41)	0.565	0.491 (0.344-0.702)	<0.001	0.904 (0.537-1.522)	0.703
IV	0.413 (0.222-0.769)	0.005	0.700 (0.31-1.579)	0.390	0.26 (0.141-0.479)	<0.001	0.438 (0.186-1.031)	0.059
Figo stage^i^								
I	Reference		Reference^j^		Reference		Reference^j^	
II	0.416 (0.169-1.022)	0.056	—	—	0.536 (0.21-1.364)	0.191	—	—
III	0.373 (0.118-1.176)	0.092	—	—	0.333 (0.105-1.054)	0.062	—	—
IV	0.639 (0.056-7.243)	0.718	—	—	0.571 (0.05-6.483)	0.652	—	—
Ann Arbor stage^j^								
I	Reference		Reference^j^		Reference		Reference^j^	
II	1.733 (0.757-3.97)	0.193	—	—	0.851 (0.286-2.534)	0.772	—	—
III	1.3 (0.468-3.614)	0.615	—	—	1.164 (0.283-4.793)	0.834	—	—
IV	1.675 (0.69-4.069)	0.255	—	—	0.506 (0.168-1.53)	0.228	—	—

Univariable: *P* < 0.1 is included in multivariable analyses. Multivariable: *P* < 0.05 is significant. Method = enter. Tolerance (value <0.1 indicates multi-collinearity), variance inflation factors (value >10 indicates multi-collinearity) and variance proportions (proportions on the same eigenvalue ≥0.7 indicate multi-collinearity) were checked for multi-collinearity.

CI, confidence interval; OR, odds ratio; n.s., not specified due to low numbers.

a Primary school, junior high school or equivalent.

b Senior high school or equivalent.

c College/university.

d Institutional households, one-parent household or any other household than already included.

e Member of institutional household, reference person in other household or any other member than already included.

f Unspecified sites, primary sites unknown or unknown tumor type and eye.

g For example general practitioner, freely established specialist or foreign hospital.

h Figo and Ann Arbor stage were included as a separate category in order to include all tumor types, but are not reported.

i Gynecological malignancies.

j Hematological malignancies.

k Variables were excluded from multivariable analysis due to univariable analysis *P* > 0.1.

l Variables were excluded from multivariable analysis due to multi-collinearity.

## Discussion

### Findings

This nationwide, population-based case-control registry study showed that AYA cancer survivors are significantly less often employed 1 and 5 years after diagnosis compared with an age- and sex-matched control population. Also, AYA cancer survivors received significantly more often disability benefits compared with controls both short and long-term. Personal and household income, however, did not significantly differ between these groups. Females were significantly more often unemployed in the long-term compared with males. Unemployed AYAs received significantly more often unemployment benefits, social assistance benefits, social security benefits and disability benefits compared with employed AYAs. Unfortunately, receiving these benefits does not compensate for differences in income: personal income was on average significantly higher among those employed for all time points compared with those unemployed. Similarly, employed AYAs were significantly more often economically independent. The results of this study are consistent with other international studies among AYA cancer survivors that show affected employment and financial outcomes.^16^^,^^27^^,^^28^^,^^32^^,^^37^^,^^38^

This study adds to the current knowledge by showing significantly decreased levels of employed AYAs compared with matched controls based on objective, registry-based data, both short- and long-term. The percentage of unemployed AYAs significantly increases from baseline (17.4%) to 1 year later (23.8%) and still remains increased 5 years after diagnosis (20.7%). This is in line with the study of Sisk and colleagues,^28^ showing increasing percentages of unemployed AYAs after their diagnosis over time and Dahl and colleagues^38^ who show a long-term (≥6 years since first cancer diagnosis) reduction in work ability among Norwegian young adult cancer survivors. Guy and colleagues^20^ also reported significant differences between AYAs and controls: 33.4% of the AYA cancer survivors were not employed versus 27.4% of the controls. Differences in results may be explained by self-reported versus objective data, types of employment included (i.e. employees and/or self-employed) and worldwide differences in health care and social security systems.^39^ Although childhood and older adult cancer survivors may also experience poor work-related outcomes, study results may not be directly comparable due to differences between populations (e.g. age at diagnosis, tumor types and related treatments, different long-term and late effects, no versus limited versus extensive work experience, and one of the first jobs versus close to retirement).^9^^,^^40^^,^^41^

The study of Teckle and colleagues^32^ among young Canadian cancer survivors indicates that AYAs may face lower income than their peers. In our study, personal and household income did not differ between the AYAs and controls, even though differences in employment were observed. This may be explained by the set of available benefits (disability, unemployment, social welfare, social security) for which one can apply in the Netherlands that may (partly) compensate for the lost income from employment.^42^ The income variables used in this study consist of, among others, income from employment as well as received benefits. This would indicate that the Dutch social support system does help AYAs well with keeping a stable income. When zooming into (un)employed AYAs, however, significant differences were seen in income even when benefits were provided. This indicates there is also a group of survivors who may not be employed because of the effects of cancer (treatment) and who have to get by with significantly less income: available financial benefits do not fully cover the difference. This, in combination with possibly having high medical costs and less financial reserves,^16^^,^^20^ may have a (long-term) financial impact on AYAs. Which factors are associated with AYAs’ financial outcomes and how this may change over time is currently being explored. In addition, it is important to have a closer look at which AYAs are staying behind and how to best provide support. It should be noted that a larger part of the unemployed AYA cancer population is studying compared with those employed short-term, which may partly explain differences in income. This is in line with literature showing that many AYAs face educational disruptions and perhaps have to change their educational goals, leading to many AYAs still studying.^6^^,^^18^^,^^43^

Based on treatment data, results showed that unemployed AYA cancer survivors 1 and 5 years after diagnosis were more often treated with systemic treatments (i.e. chemotherapy, radiotherapy, hormone therapy). This indicates that a heavier treatment burden may significantly impact employment outcomes, also in the long-term. These findings are in line with the study of Dahl and colleagues,^38^ where both low work ability and non-employment were associated with heavier treatment burden. This result was not observed within our multivariable logistic regression analyses, however, except for chemotherapy. A cancer diagnosis of the CNS was also associated with both short- and long-term unemployment. This is in line with results of other studies and can be explained by the cognitive and/or physical problems often experienced by CNS cancer survivors.^19^^,^^44^

Findings of this study also indicate an impact on household level, reinforcing current knowledge that not only the AYA cancer survivor him/herself is impacted, but also their close ones. Ketterl and colleagues^37^ showed that some families borrow money, incur debt or even file for bankruptcy because of the cancer (treatment), which negatively affects their prospects. To what extent this takes place may differ per country and health care system. Future research should assess the degree of impact of an AYA cancer diagnosis on a household level.

As the current study included objective, population-based registry data only, we do not know any specific reasoning for unemployment based on patient-reported outcomes (PROs). Some AYAs may be forced due to the adverse effects of their cancer (treatment) or may face employment-related discrimination, whereas others may deliberately choose to change their employment status, because of their changed perspective on life and employment due to their experience.^18^^,^^27^^,^^37^^,^^38^^,^^45^ So far, less is known about how often AYAs face employment-related discrimination.^27^ Fortunately, there is also a group of AYAs who are doing well: they do not experience any objective work and financial issues. It is unknown, however, whether they made use of support systems, like AYA-specific health care for example. Furthermore, although their objective outcomes are good, it can be the case that these AYAs still experience subjective work and financial issues not captured by objective measures. Further research, in which objective data and PROs are combined, is needed to evaluate the objective study results in light of the personal situation and perspective of the AYA him/herself.

### Strengths and limitations

Many studies carried out in the past have used self-reported data only, possibly leading to biased results. Our study’s strength is the use of objective, nationwide, population-based registry data. Also, the inclusion of a control population made it possible to compare employment and financial outcomes with age- and sex-matched controls, which has been done only to a limited extent up until now.^32^ In addition, whereas many studies focus on one invasive tumor type or a subset of invasive tumor types, in this study all invasive tumor types were included improving the generalizability of the results.

A limitation may be that the inclusion criteria for the cancer survivor sample were not exactly the same as for the controls. AYAs had to be alive for 5 years after diagnosis to be included, whereas the controls had to be living in the Netherlands with a known source of income for the same span of 5 years in order to be included (i.e. prevent inclusion from those emigrating). Comparable choices were made in the study of de Wind and colleagues^46^ who argue, however, that this has most likely not impacted study results. Secondly, of all AYAs, 4.4% could not be linked with their CBS ID. Significant differences were seen between AYAs who were linked with their CBS ID and those not linked, regarding age, several therapies and the type of hospital of treatment. As it concerns only a very small percentage of the total population, however, we do not expect major differences regarding employment and financial outcomes. Also, what the effects are of new treatments, including immunotherapy and targeted therapy, are unknown as this study was done in a population where these therapies have largely not yet been introduced. Lastly, as mentioned earlier, we have no subjective data that may help interpreting current objective results based on AYAs’ cancer experience.

### Recommendations for further research and practice

Taking into account the results of this study, future research is needed that combines objective data and PROs to understand the self-reported reasons for the (objective) employment and financial outcomes, especially for those at risk of decreased income levels, economic dependence and unemployment, and what we can learn from those who do not experience any issues. As an extension, relatives/households could be part of these studies as well, since the effect of a diagnosis is not limited to the patient him/herself. Also, gaining knowledge about how to best help those at increased risk of adverse outcomes with supportive, age-specific interventions (i.e. type of support, time wise, involvement of stakeholders like clinical occupational physicians, available tools and resources) may help improving AYA cancer survivorship outcomes. These interventions should be incorporated into AYA programs, focusing on age-specific instead of tumor-specific aspects. The entire population of AYAs should be taken into account as issues and rationales may differ—e.g. those with an uncertain or poor prognosis may value employment as much as those diagnosed with a lower stage. Study results provide input for developing AYA-specific practical interventions that are part of standard AYA survivorship care, including creating awareness and knowledge of possible short- and long-term disease-related effects, providing education and structured guidance and the availability of resources and support. To properly embed all of this, both guidelines and policies must be drawn up to guarantee the existence and quality of AYA specific survivorship care focusing on employment and financial outcomes.

### Conclusion

Using objective, nationwide, population-based registry data, the results of this study show that AYA cancer survivors are more often unemployed compared with age- and sex-matched controls, up to at least 5 years after diagnosis. Although financial benefits are widely available, those unemployed have a significantly lower income than employed AYAs. Therefore, actions should be taken to address the employment and financial outcomes of AYA cancer survivors at the level of cancer care institutions proactively providing tailored cancer survivorship care, as well as on a macro system level where policies and programs should be developed. Providing support regarding employment and financial outcomes from diagnosis onwards may help AYAs finding their way (back) into society.
