# Supplementary material for: Short- and long-term impact of cancer on employment and financial outcomes of adolescents and young adults (AYAs): a large population-based case-control registry study in the Netherlands
Source: ESMO Open. 2022 Jun 27;7(4):100521. doi: 10.1016/j.esmoop.2022.100521 (PMC9434129; doi:10.1016/j.esmoop.2022.100521)
Supplement: Supplementary Material [file mmc1.docx]

**Supplementary materials**

**Table S1. Characteristics of the AYA cancer patients who were linked with their CBS ID and those who were not.**

|  |  | **Linked AYAs**  **(N=2528)** | **Non-linked AYAs (N=117)** |  |
| --- | --- | --- | --- | --- |
|  |  | **N (%)** | **N (%)** | **p-value** |
| **Sex** | **Male** | 1068 (42.2) | 53 (45.3) | 0.514 |
|  | **Female** | 1460 (57.8) | 64 (54.7) |  |
| **Age at diagnosis (mean (SD))** | | 31.7 (5.7) | 29.0 (6.0) | <0.001 |
| **Organ surgery** | **No** | 1149 (45.5) | 66 (56.4) | 0.020 |
|  | **Yes** | 1379 (54.5) | 51 (43.6) |  |
| **Local surgery** | **No** | 1813 (71.7) | 78 (66.7) | 0.237 |
|  | **Yes** | 715 (28.3) | 39 (33.3) |  |
| **Chemotherapy** | **No** | 1472 (58.2) | 86 (73.5) | 0.001 |
|  | **Yes** | 1056 (41.8) | 31 (26.5) |  |
| **Radiotherapy** | **No** | 1781 (70.5) | 90 (76.9) | 0.133 |
|  | **Yes** | 747 (29.5) | 27 (23.1) |  |
| **Hormone therapy** | **No** | 2212 (87.5) | ≥90% | 0.008 |
|  | **Yes** | 316 (12.5) | ≤10% |  |
| **Type of cancer** | **Bone, articular cartilage and soft tissues** | 72 (2.8) | ≤10 | n.a. |
|  | **Breast** | 508 (20.1) | ≤10 |  |
|  | **Central nervous system** | 80 (3.2) | ≤10 |  |
|  | **Digestive tract** | 120 (4.7) | ≤10 |  |
|  | **Endocrine glands** | 124 (4.9) | ≤10 |  |
|  | **Female genital organs** | 201 (8.0) | 10 (8.5) |  |
|  | **Hematological malignancies** | 339 (13.4) | 19 (16.2) |  |
|  | **Head and neck** | 46 (1.8) | ≤10 |  |
|  | **Male genital organs** | 444 (17.6) | 20 (17.1) |  |
|  | **Respiratory tract** | 23 (0.9) | ≤10 |  |
|  | **Skin** | 510 (20.2) | 26 (22.2) |  |
|  | **Urinary tract** | 51 (2.0) | ≤10 |  |
|  | **Other/unspecified sites^1^** | 10 (0.4) | ≤10 |  |
| **Type of hospital of treatment** | **University hospital** | 645 (25.5) | 47 (40.2) | <0.001 |
|  | **General/ Collaborating top clinical hospital or other^2^** | 1883 (74.5) | 70 (59.8) |  |
| **Stage^3^** | **I** | 1173 (59.0) | 57 (64.8) | 0.129 |
|  | **II** | 434 (21.8) | 14 (15.9) |  |
|  | **III** | 206 (10.4) | ≤10 |  |
|  | **IV** | 47 (2.4) | ≤10 |  |
|  | **Missing** | 128 (6.4) | 12 (13.6) |  |
| **Figo stage ^4^** |  | 201 (100.0) | 10 (100.0) |  |
| **Ann arbor stage^5^** |  | 339 (100.0) | 19 (100.0) |  |
| ^1^ Unspecified sites, primary sites unknown or unknown tumor type and eye. ^2^ E.g. general practitioner, freely established specialist or foreign hospital. ^3^ Figo and Ann arbor stage were not included in the Stage variable. ^4^ Gynecological malignancies; frequencies could not be displayed due to disclosure guidelines of CBS. ^5^ Hematological malignancies; frequencies could not be displayed due to disclosure guidelines of CBS. n.a.: not applicable due to low numbers. Note: frequencies ≤10 could not be displayed due to disclosure guidelines of CBS. Independent samples t-tests and Chi-square tests were performed to compare those linked with their CBS ID with those not linked. | | | | |
